# Regulation of K^+^ Conductance by a Hydrogen Bond in Kv2.1, Kv2.2, and Kv1.2 Channels

**DOI:** 10.3390/membranes11030190

**Published:** 2021-03-09

**Authors:** Yuchen Zhang, Xuefeng Zhang, Cuiyun Liu, Changlong Hu

**Affiliations:** Department of Physiology and Biophysics, Institutes of Brain Science, School of Life Sciences, Fudan University, Shanghai 200032, China; 19110700020@fudan.edu.cn (Y.Z.); 18210700080@fudan.edu.cn (X.Z.); 15210700016@fudan.edu.cn (C.L.)

**Keywords:** voltage-gated potassium channels, channel inactivation, hydrogen bonds, potassium conductance

## Abstract

The slow inactivation of voltage-gated potassium (Kv) channels plays an important role in controlling cellular excitability. Recently, the two hydrogen bonds (H-bonds) formed by W434-D447 and T439-Y445 have been reported to control the slow inactivation in *Shaker* potassium channels. The four residues are highly conserved among Kv channels. Our objective was to find the roles of the two H-bonds in controlling the slow inactivation of mammalian Kv2.1, Kv2.2, and Kv1.2 channels by point mutation and patch-clamp recording studies. We found that mutations of the residues equivalent to W434 and T439 in *Shaker* did not change the slow inactivation of the Kv2.1, Kv2.2, and Kv1.2 channels. Surprisingly, breaking of the inter-subunit H-bond formed by W366 and Y376 (Kv2.1 numbering) by various mutations resulted in the complete loss of K^+^ conductance of the three Kv channels. In conclusion, we found differences in the H-bonds controlling the slow inactivation of the mammalian Kv channels and *Shaker* channels. Our data provided the first evidence, to our knowledge, that the inter-subunit H-bond formed by W366 and Y376 plays an important role in regulating the K^+^ conductance of mammalian Kv2.1, Kv2.2, and Kv1.2 channels.

## 1. Introduction

Mammalian voltage-gated potassium (Kv) channels, consisting of 12 subfamilies (Kv1-12), including 40 subtypes, play key roles in maintaining the membrane potential and mediating neuronal and muscular excitability and have been implicated in various neuronal and cardiovascular diseases [1,2,3]. Kv channels are tetramers forming a K^+^-selective pore through four α-subunits. Each α-subunit comprises six transmembrane helices (S1-S6) and a re-entrant P loop between S5 and S6, which includes a pore helix and a selectivity filter. Kv channels commonly open at depolarization and enter an inactivated state under prolonged depolarization, which blocks K^+^ conduction and plays a key role in controlling cellular excitability.

The inactivation of Kv channels includes two distinct processes, termed N-type (fast) and C-type (slow) inactivation [4]. Slow inactivation is presumably associated with the conformational changes in the selectivity filter region, which can be influenced by the pore-helix residues or the adjacent extracellular vestibule [5,6,7,8]. The mechanism underling the slow inactivation of Kv channels has been intensively studied on the bacterial KcsA and *Drosophila* shaker channels (in the following text, *shaker* stands for the *Drosophila* shaker channel.). Hydrogen bonds (H-bonds) between the selectivity filter and the adjacent pore helix are considered as major regulators of the slow inactivation. The two H-bonds formed by W67-D80 and E71-D80 have been shown to play key roles in controlling the slow inactivation of KcsA channels [9,10]. The W67 and D80 are highly conserved in Kv channels. Mutations at equivalent positions in *Shaker* channels (W434 and D447) lead to drastic changes in slow inactivation [11,12,13,14]. Using synthetic amino acids, Pless et al. recently demonstrated that an intra-subunit H-bond formed by W434-D447 and an inter-subunit H-bond formed by T439-Y445 regulate the slow inactivation of *Shaker* channels [15]. Besides these four residues, T449 is also shown to play important roles in regulating the slow inactivation of *Shaker* channels [16]. However, mutations at this site do not result in similar effects on the slow inactivation of mammalian Kv1.4 and Kv1.5 channels [17,18], which suggests that the residues controlling slow inactivation for mammalian Kv channels and *Shaker* channels may be different. Compared to *Shaker* channels, the role of the H-bonds in mammalian Kv channels is seldom reported. The four residues, forming the two H-bonds (W434-D447 and T439-Y445) in *Shaker* channels, are highly conserved among the Kv channels and may also form H-bonds in mammalian Kv channels according to the structure of the Kv1.2/Kv2.1 chimeric channel (PDB: 2R9R).

In this study, we tested whether the two H-bonds (W434-D447 and T439-Y445) in the *Shaker* channels are the key regulators of the slow inactivation for mammalian Kv2.1, Kv2.2, and Kv1.2 channels by point mutation and patch-clamp recording studies.

## 2. Materials and Methods

### 2.1. Cell Culture and Transfection

Human embryonic kidney cells (HEK293) were purchased from the cell bank of the Chinese Academy of Sciences (Shanghai, China). Cells were cultured in Dulbecco’s modified Eagle’s medium (GIBICO, Grand Island, NY, USA) supplemented with 10% fetal bovine serum and 1% antibiotic-antimycotic solution at 37 °C with 5% CO_2_. Plasmids for rat wild-type and mutant Kv channels were transiently transfected using jetPRIME reagents (Polyplus transfection) according to the manufacturer’s instructions. The cells were used for patching experiments 24h after transfection.

### 2.2. Molecular Biology

Wild-type rat Kv1.2 (NM_012970.3), Kv2.1 (NM_013186.1), and Kv2.2 (NM_054000.2) cDNAs were subcloned into pEGFPN1 expression vectors. Site-directed mutagenesis for these Kv channels was achieved by using the Quick-Change XL Site-directed Mutagenesis kit (Stratagene, La Jolla, CA, USA). All mutations were confirmed by sequencing.

### 2.3. Electrophysiology

Whole-cell currents in HEK293 were recorded using an Axopatch 200B amplifier (Axon Instruments, Union City, CA, USA) operated in voltage-clamp mode and data collected with PCLAMP 10.7 software (Axon Instruments). The bath solution contained (in mM): 140 NaCl, 2.5 KCl, 2.5 CaCl_2_, 10 glucose, 10 HEPES, and 1 MgCl_2_ (pH adjusted to 7.4 using NaOH). The internal solution contained (in mM): 135 potassium gluconate, 10 KCl, 1 CaCl_2_, 1 MgCl_2_, 10 HEPES, 2 Mg-ATP, and 10 EGTA (pH adjusted to 7.3 using KOH). The pipettes were created from capillary tubing (BRAND, Wertheim, Germany) and had resistances of 4 to 6 MΩ under these solution conditions. Currents were sampled at 10 kHz and filtered at 2 kHz and corrected online for leak and residual capacitance transients using a P/4 protocol. Data from cells with seal resistance > 1 GΩ and series resistance < 9 MΩ were included in this study. Series resistance was compensated to 70% (10-s lag). All of the recordings were performed at room temperature.

### 2.4. Western Blot

Proteins on the surface of HEK293 cells transfected with the wild-type and mutant Kv channels were biotin-labeled and isolated using a Pierce cell surface protein biotinylation and isolation kit (#A44390, Thermo Scientific, Rockford, IL, USA) according to the manufacturer’s instructions. An aliquot of the cell lysate was kept as the total protein fraction. The protein samples were resolved using 10% SDS PAGE and transferred to polyvinyldifluoride membranes (Millipore, Burlington, MA, USA) in a transfer buffer (25-mM Tris, 192-mM glycine, and 20% methanol, *v*/*v*) at 100 V for 1 h. The membranes were blocked with 10% nonfat dry milk in TBST for 1 h at room temperature. The membranes were incubated with the primary antibody (Anti-Kv1.2, 1:1000 (#75-008, NeuroMab, Davis, CA, USA); Anti-Kv2.1, 1:500 (#75-014, NeuroMab); Anti-Kv2.2, 1:1000 (#APC120AN0150, Alomone Labs, Jerusalem, Israel); and anti-beta tubulin, 1:5000 (#M20005, Abmart, Shanghai, China)) in an Immunoreaction Enhancer Solution for the primary antibody (TOYOBO, Osaka, Japan) overnight at 4 °C. The membranes were washed 3 times for 10 min in TBST and incubated with a horseradish peroxidase-conjugated secondary antibody (1:10,000) (Beyotime, Shanghai, China) in TBST for 2 h at room temperature. The blots were developed using enhanced chemiluminescence reagents and the ChemiDoc XRS+imaging system from Bio-Rad (Hercules, CA, USA).

### 2.5. Statistics

Data analysis was performed with Clampfit 10.2 (Axon Instruments) and Origin 8.0 software (Origin Lab, Northampton, MA, USA). Statistical analysis consisted of unpaired or paired Student’s *t*-tests. Data are given as means ± SEM, and *n* indicates the number of tested cells or independent tests. *p* < 0.05 was considered statistically significant.

## 3. Results

### 3.1. Mutation of the Residues Equivalent to W434 and T439 in Shaker Channels Did Not Change Slow Inactivation of Kv2.1, Kv2.2, and Kv1.2 Channels

**Figure 1:**
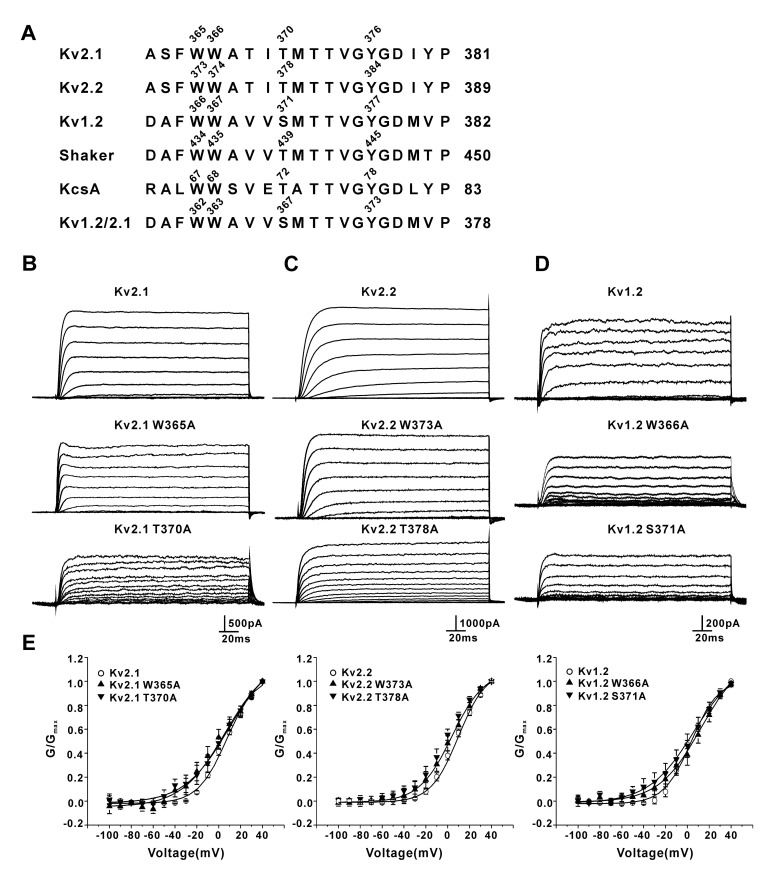
Effects of the mutation of the equivalent residues controlling the slow inactivation of *Shaker* channels on the rat Kv2.1, Kv2.2, and Kv1.2 channels. (**A**) Sequence alignment of the selectivity filter and pore helix of various K+ channels: Kv2.1 (GI: 6981120), Kv2.2 (GI: 164663795), Kv1.2 (GI: 25742772), *Shaker* (GI: 288442), KcsA (GI: 61226909), and the Kv1.2/Kv2.1 chimeric channel (PDB: 2R9R). (**B**) Representative currents for the Kv2.1 wild-type, W365A, and T370A mutant channels (−100 to +40 mV, 200 ms, in 10-mV increments; *n* = 8~14). (**C**) Representative currents for the Kv2.2 wild-type, W373A, and T378A mutant channels (*n* = 7~12). (**D**) Representative currents for the Kv1.2 wild-type, W366A, and S371A mutant channels (*n* = 6–9). (**E**) G-V curves of the wild-type and mutant Kv channels.

The four residues, forming two H-bonds (W434-D447 and T439-Y445) in *Shaker* channels, were conserved among voltage-gated potassium channels. The residue numbers for each channel are shown in Figure 1A. We conducted mutations for two of them in the Kv2.1, Kv2.2, and Kv1.2 channels. As shown in Figure 1B, the W365A and T370A mutations (equivalent to W434 and T439 in *Shaker*) did not change the slow inactivation of the Kv2.1 channel overexpressed in HEK293 cells. K^+^ currents were elicited by 200-ms steps (−100 to +40 mV in 10-mV increments) applied every 10 s from a holding potential of −100 mV. The slow inactivation of the Kv2.2 channel was not altered by either W373A or T378A mutations (Figure 1C). The W366A and S371A mutations (Kv1.2 numbering) did not alter the slow inactivation of the Kv1.2 channel overexpressed in HEK293 cells (Figure 1D). The steady-state activation curve (G-V) of the Kv2.1, Kv2.2, and Kv1.2 currents were obtained by plotting the normalized conductance (G/G_max_) as a function of the command voltage (Figure 1E). The half-activation potentials (V_1/2_) of the Kv channels were obtained by fitting the conductance with the Boltzmann function. The half-activation potentials (V_1/2_) of Kv2.1, Kv2.2, and Kv1.2 were not significantly changed by the mutations (V_1/2_:Kv2.1, 7.8 ± 2.1 mV, Kv2.1W365A, 3.8 ± 7.4 mV, and Kv2.1T370A, 4.4 ± 3.7 mV, *n* = 7–11, *p* > 0.05, wild type (WT) versus mutants; Kv2.2, 5.7 ± 1.9 mV, Kv2.2W373A, 1.7 ± 3.7 mV, and Kv2.2T378A, 2.1 ± 5.7 mV, *n* = 8–14, *p* > 0.05, WT versus mutants; and Kv1.2, 3.2 ± 2.2 mV, Kv1.2W366A, 9.1 ± 4.1 mV, and Kv1.2S371A, 6.7 ± 4.9 mV, *n* = 6–9, *p* > 0.05, WT versus mutants. Slope of activation: Kv2.1, 13.0 ± 0.6 mV, Kv2.1W365A, 16.3 ± 2.9 mV, and Kv2.1T370A, 17.2 ± 4.9 mV, *n* = 7–11, *p* > 0.05, WT versus mutants; Kv2.2, 12.1 ± 0.4 mV, Kv2.2W373A, 13.6 ± 0.9 mV, and Kv2.2T378A, 11.6 ± 1.1 mV, *n* = 8–14, *p* > 0.05, WT versus mutants; and Kv1.2, 11.5 ± 0.7 mV, Kv1.2W366A, 17.5 ± 3.1 mV, and Kv1.2S371A, 18.5 ± 5.0 mV, *n* = 6–9, *p* > 0.05, WT versus mutants). The Kv2.1, Kv2.2, and Kv1.2 channels are slow-inactivating; therefore, longer depolarizing pulse (5s) was used to further test the effects of the mutations on the inactivation.

**Figure 2:**
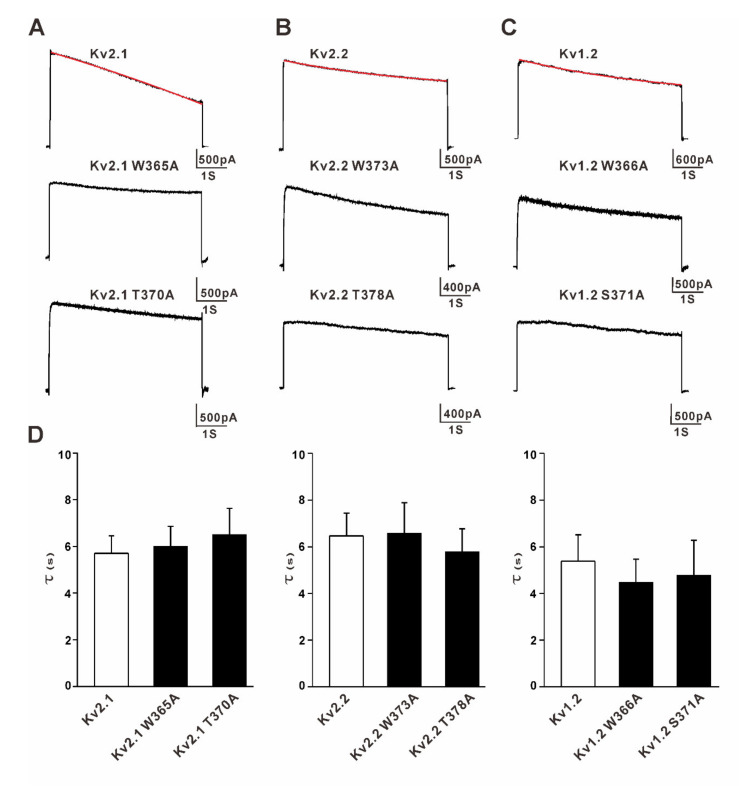
Effects of the mutation of the time constants of the inactivation of the Kv2.1, Kv2.2, and Kv1.2 channels. The inactivation time constants for the wild-type and mutant Kv channels were determined by fitting a 5-s depolarization at +20 mV with single exponentials. Red lines show the representative fitting trajectories of the current traces. (**A**). Representative currents for the Kv2.1 wild-type, W365A, and T370A mutant channels (5-s pulses from −100 mV to 20 mV) (**B**). Representative currents for the Kv2.2 wild-type, W373A, and T378A mutant channels. (**C**) Representative currents for the Kv1.2 wild-type, W366A, and S371A mutant channels. (**D**) Comparison of the time constants of the inactivation of the wild-type and mutant Kv channels, *n* = 6~12, *p* > 0.05 (wild type versus mutants).

The inactivation time constants for the wild-type (WT) and mutant Kv channels were determined by fitting a 5-s depolarization at +20 mV with single exponentials. The W365A and T370A mutations (Kv2.1 numbering) did not change the slow inactivation of the Kv2.1, Kv2.2, and Kv1.2 channels overexpressed in HEK293 cells (inactivation of Kv2.1, 5.6 ± 1.0 s, Kv2.1W365A, 6.0 ± 1.3 s, and Kv2.1T370A, 6.5 ± 1.7 s, *n* = 6 to 7, *p* > 0.05, WT versus mutants; Kv2.2, 6.4 ± 1.6 s, Kv2.2W373A, 6.6 ± 2.2 s, and Kv2.2T378A, 5.8 ± 1.2 s, *n* = 6–10, *p* > 0.05, WT versus mutants; and Kv1.2, 5.3 ± 1.4 s, Kv1.2W366A, 4.5 ± 1.0 s, and Kv1.2S371A, 4.8 ± 2.1 s, *n* = 6–9, *p* > 0.05, WT versus mutants; Figure 2). The data above indicated that the two H-bonds (W434-D447 and T439-Y445) in the *Shaker* channels are not the key regulators of the slow inactivation for the mammalian Kv2.1, Kv2.2, and Kv1.2 channels.

### 3.2. Mutation of a Conserved Tryptophan Residue Resulted in the Loss of Function of the Kv2.1, Kv2.2, and Kv1.2 Channels in HEK293 Cells

**Figure 3:**
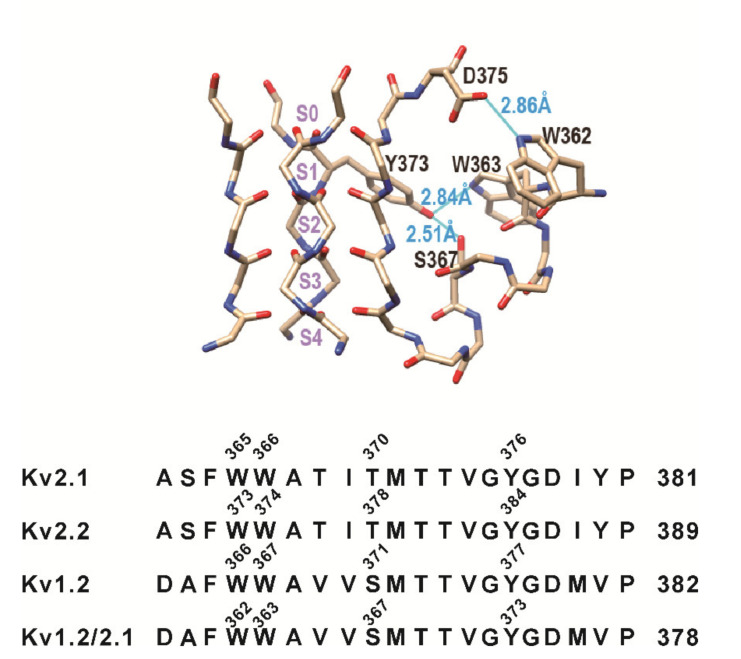
Selectivity filter structure of the Kv1.2/Kv2.1 chimeric channel (PDB: 2R9R) with the ion-binding sites S0–S4 labeled. The relevant pore helix residues and the three putative hydrogen bonds (blue lines) are labeled as well.

Structural evidence [19,20] suggests that W366 and Y376 (Kv2.1 numbering) may form an inter-subunit hydrogen bond besides the two H-bonds tested above. The structure of the Kv1.2/Kv2.1 chimeric channel (PDB: 2R9R) shows three putative hydrogen bonds formed by these residues (Figure 3). The Kv1.2/Kv2.1 chimeric channel is derived from a rat Kv1.2 K^+^ channel. It was constructed by replacing the S3b and S4 helices of the Kv1.2 channel with the corresponding voltage-sensor region of the Kv2.1 channel [20]. Therefore, the sequence numbering in the chimeric channel was slightly different from that of the Kv1.2 channel. However, the pore region of the chimeric channel was the same as that of the Kv1.2 channel.

The crystal structure shows that W362 can form a hydrogen bond with D375, a residue at the top of the selectivity filter, and W363 and S367 each may form a hydrogen bond with Y373, a residue in the upper portion of the selectivity filter. Thus, they can exert influences on the selectivity filter via these hydrogen bonds. The mutations of these residues may affect the stability, or even the conformation, of the selectivity filter.

**Figure 4:**
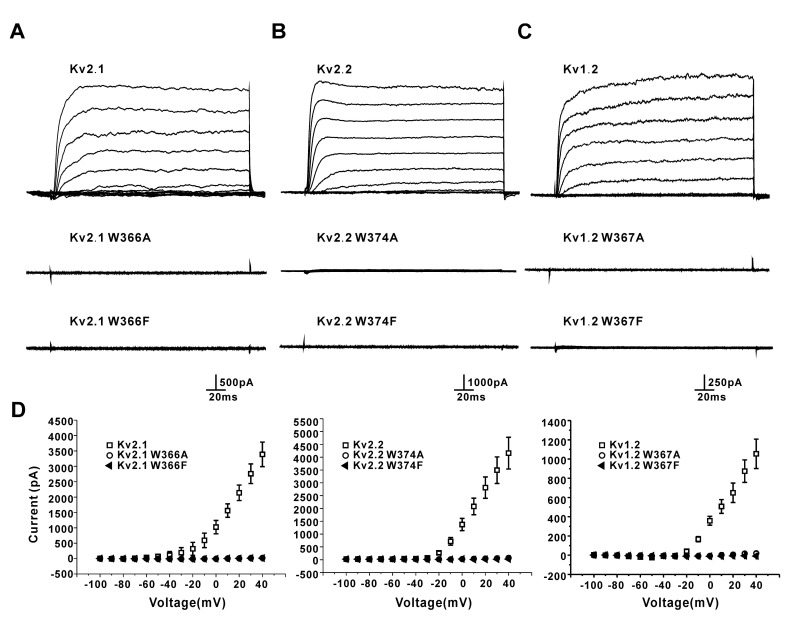
Effects of the mutations of the conserved tryptophan residue on the Kv2.1, Kv2.2, and Kv1.2 channel currents in HEK293 cells. (**A**) Representative currents for the Kv2.1 wild-type, W366A, and W366F mutant channels. (**B**) Representative currents for the Kv2.2 wild-type, W374A, and W374F mutant channels. (**C**) Representative currents for the Kv1.2 wild-type, W367A, and W367F mutant channels. (**D**) I-V plots of the outward currents of the Kv2.1, Kv2.2, and Kv1.2 wild-type and mutant channels (*n* = 7–14).

We tested the effect of the H-bond (W366-Y376) by the W366A mutation in the Kv2.1 channel. Surprisingly, HEK293 cells transfected with the mutant channels presented no outward potassium currents (Figure 4A), indicating the complete loss of K^+^ conductance or permanent slow inactivation. Next, the W366F mutation was used to test whether the aromatic side chain was involved in this change. As shown in Figure 4A, the W366F mutation had a similar effect on the Kv2.1 channel as W366A did, which suggested that the loss of function might be due to the loss of the H-bond between W366 and Y376. Then, we did similar mutations for the Kv2.2 and Kv1.2 channels. As shown in Figure 4B,C, the W374A/F (Kv2.2) and W367A/F (Kv1.2) mutations produced similar effects. 

**Figure 5:**
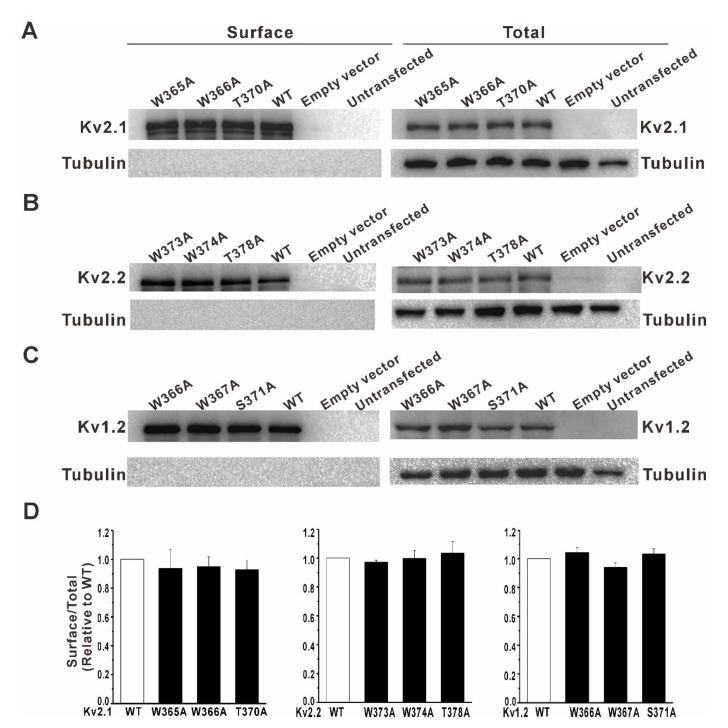
Cell surface expression of the various Kv channels in HEK293 cells. The Kv channel surface expression was detected using the cell surface biotinylation of HEK293 cells transfected with the wild-type/mutant Kv channels. The left panels were the biotinylated membrane protein samples, and the right panels were total protein samples. (**A**) Sample images showing endogenous or transfected wild-type/mutant Kv2.1 channel expression. (**B**) Sample images showing endogenous or transfected wild-type/mutant Kv2.2 channel expression. (**C**) Sample images showing endogenous or transfected wild-type/mutant Kv1.2 channel expression. The signals of the intracellular protein beta-tubulin were detected only in the total protein samples (right panels) and not in the biotinylated surface protein samples (left panels). Endogenous cell surface protein expression of the Kv2.1, Kv2.2, and Kv1.2 channels was undetectable. (**D**) The mutations did not alter the cell surface expression of the Kv channels in HEK293 cells (*n* = 3, *p* > 0.05, WT versus mutants).

The mutant channels showed no obvious outward potassium currents, which might be caused by the point mutation interfering with the protein expression or trafficking to the cell surface. Therefore, we tested the cell surface expression of the various Kv channels in HEK293 cells. As shown in Figure 5, HEK293 cells do not endogenously express the Kv2.1, Kv2.2, and Kv1.2 channels. In the transfected HEK293 cells, whether with wild-type (WT) or mutant Kv2.1, Kv2.2, and Kv1.2 channels, the surface expression of the Kv channel was clearly detected, and no significant difference was found between the wild-type and mutant channels, indicating that the complete loss of K^+^ conductance shown in Figure 3 was not due to the change of the channel protein expression or the failure of trafficking to the cell surface.

### 3.3. Y376F (Kv2.1), Y384F (Kv2.2), and Y377F (Kv1.2) Mutations Resulted in Complete Loss of K^+^ Conductance of the Kv Channels Expressed in the HEK293 Cells

**Figure 6:**
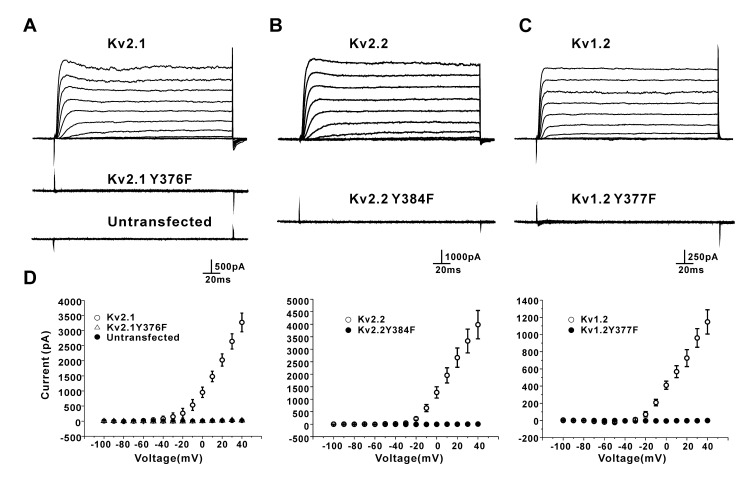
Effects of the mutation of the conserved tyrosine residue on the Kv2.1, Kv2.2, and Kv1.2 channels in HEK293 cells. (**A**) Y376F (Kv2.1) mutant channels present no obvious outward currents. HEK293 cells present no obvious endogenous outward currents. (**B**,**C**) Y384F (Kv2.2) and Y377F (Kv1.2) mutant channels present no obvious outward currents. Currents are elicited by a step protocol (holding at −100 mV and depolarizing in 10-mV steps from −100 to +40 mV at 10-s intervals). (**D**) I-V plots of the outward currents of Kv2.1, Kv2.2, and Kv1.2 wild-type and mutant channels (*n* = 5~13).

If W366 and Y376 form an H-bond that controls the K^+^ conductance of the Kv2.1 channel, the mutation of Y376 should mimic the effect of the W366F mutation. As shown in Figure 6, the Y376F mutant channels indeed showed no potassium current, same as the W366F mutant channels. The Y384F and Y377F mutations resulted in similar effects on the Kv2.2 and Kv1.2 channels, respectively (Figure 6). These data indicate that W366 and Y376 may form an H-bond that controls the K^+^ conductance of mammalian Kv channels.

## 4. Discussion

In this study, we tested whether the H-bonds controlling the slow inactivation in *Shaker* channels play important roles in the rat Kv2.1, Kv2.2, and Kv1.2 channels in HEK293 cells. The W365 and D378 residues (Kv2.1 numbering) are highly conserved in Kv channels. The H-bond formed by these two residues plays key roles in controlling the slow inactivation of *Shaker* channels. However, disrupting the H-bond by point mutation of tryptophan to alanine showed little effect on the slow inactivation in the mammalian Kv2.1, Kv2.2, and Kv1.2 channels expressed in HEK293 cells. Moreover, we checked other electrophysiological properties of these WT and mutant channels; the activation properties of the Kv1.2 and Kv2.1 channels in this study were similar to the previous reported recordings of Kv1.2 and Kv2.1 overexpressed in HEK293 cells [21,22,23], and, again, no significant differences were found between the WT and the W365A and T370A (Kv2.1) or W366A and S371A (Kv1.2) mutations.

Even though HEK293 cells are a commonly used model system for studying exogenous voltage-gated potassium channels because they have a “null” background, both endogenous voltage-gated K+ channels [24] and Na+ channels [25] in HEK293 cells have been reported previously. The observation of different Kv expressions in HEK293 may come from different culture conditions or sources of the cells in different labs. We used only a low passage number (<30) of HEK293 cells in the present study. We found very little native K^+^ currents in the HEK293 cells, with occasionally transient tiny sodium currents. Our recordings for the native K^+^ currents in the HEK 293 cells were similar to a previous paper [26], which showed that HEK 293 cells with a low passage number (<45) lack endogenous K^+^ channel activity. The cell surface expression of the Kv2.1, Kv2.2, and Kv1.2 channels was not detected in the nontransfected HEK293 cells, which excluded possible interference by the endogenous K^+^ channels. 

Compelling studies have shown that slow inactivation and ionic selectivity are functionally coupled in Kv channels. The *Shaker* and Kv2.1 channels displayed decreased K^+^ and increased Na^+^ permeability during the slow inactivation process [27,28]. The W434F mutation in the *Shaker* channel resulted in the complete loss of K^+^ conductance due to a permanent slow inactivation [11,12] but enabled Na^+^ permeation under an unphysiological condition (in the absence of internal K^+^) [12,29]. The W435F mutation had no effect on the slow inactivation of *Shaker* channels [15]. However, in this study, the W366F mutation (equivalent to W435F in *Shaker* channels) in the Kv2.1 channel resulted in no outward K^+^ currents, indicating a complete loss of K^+^ conductance, which suggests that residues controlling the slow inactivation of mammalian Kv channels and *Shaker* channels are different. The Y376F mutation had a similar effect on the Kv2.1 channels as the W366F mutation does. This similar effect occurs in the Kv2.2 and Kv1.2 mutant channels, which indicate that the inter-subunit H-bond formed by W366 and Y376 (by Kv2.1 numbering) plays an important role in the K^+^ permeation of mammalian Kv channels. Our data did not rule out the possibility that D378 (in Kv2.1 numbering) may play an important role in the slow inactivation of mammalian channels. Besides Y376 and W366 may also form an H-bond with D378, which is worth further investigation.

The selectivity filter is considered as an inactivation gate controlling ion permeation due to its flexibility and its putative conformational changes [5,25,30,31]. In addition, the pore helix residues can also interact with the selectivity filter and influence slow inactivation [7,11,12,15]. Based on the crystal structure, the disruption of the W366-Y376 H-bond may lead to an unstable filter or even trigger a conformational change of the selectivity filter. The collapsed filter conformation of the KcsA channel possibly occurs in inactivated channels [8]. Likewise, the W366-Y376 H-bond may influence the filter stability. The absence of potassium currents in the mutant channels may result from the instability of the selectivity filter.

In conclusion, we showed, for the first time, that the inter-subunit H-bond formed by W366 and Y376 (by Kv2.1 numbering) plays an important role in the K^+^ conductance of the mammalian Kv2.1, Kv2.2, and Kv1.2 channels.

## Data Availability

All data are contained within this article.
